# The nondestructive measurement of strain distributions in air plasma sprayed thermal barrier coatings as a function of depth from entire Debye–Scherrer rings

**DOI:** 10.1107/S1600576719016327

**Published:** 2020-02-01

**Authors:** Chun Li, Ping Xiao, Robert Cernik

**Affiliations:** a School of Materials, University of Manchester, Oxford Road, Manchester M13 9PL, UK

**Keywords:** thermal barrier coatings, turbine blades, nondestructive testing, high-energy synchrotron, 2D X-ray diffraction strain mapping

## Abstract

The residual strain distribution has been measured as a function of depth in both top coat and bond coat in as-received and heat-treated air plasma sprayed thermal barrier coating samples. High-energy synchrotron X-ray beams were used in transmission to produce full Debye–Scherrer rings whose non-circular aspect ratio gave the in-plane and out-of-plane strains far more efficiently than the sin^2^ψ method. The residual strain in the bond coat is found to be tensile and the strain in the β phase of the as-received sample was measured. The residual strains observed in the top coat were generally compressive (increasing towards the interface), with two kinds of nonlinear trend. These was a ‘jump’ feature near the interface, and in some cases there was another ‘jump’ feature near the surface. It is shown how these trend differences can be correlated to cracks in the coating.

## Abstract 2

A 2D method employing whole (or part) Debye–Scherrer rings has been used to obtain a more accurate and reliable strain-mapping result for high-density thermal barrier coatings. The method has other applications, but the use of very high energy synchrotron light makes the study of a highly anisotropic microstructure more tractable.

## 1. Introduction   

Thermal barrier coatings (TBCs) (Zhao & Xiao, 2009 ▸; Padture *et al.*, 2002 ▸; Clarke & Levi, 2003 ▸) are a type of ceramic coating applied to the hottest parts of turbine blades in jet engines to increase the operation temperature of the engines and thus improve their efficiency. These coatings (the top coat) are usually made of yttria stabilized zirconia (7–8 wt% Y_2_O_3_) (Clarke *et al.*, 2012 ▸) and are applied over a bond-coat layer to increase adhesion with the substrate, which is usually made of Ni superalloy. During use, a thin layer of thermally grown oxide (TGO) (usually alumina) grows on the bond coat, inhibiting further oxidation. TBC failures could lead to catastrophic component failure. However, these failure mechanisms are still not clear (Zhao & Xiao, 2009 ▸).

The failure of air plasma sprayed (APS) TBCs usually occurs at the interface region between the top and the bond coat, and it is generally believed that residual stresses in the coating are the driving forces for the failure (Evans *et al.*, 2001 ▸). Most reported research on the residual stress distribution in TBCs has been by modelling, which allows the stress distribution as a function of depth in the coating to be simulated. However, the microstructure of a TBC is very complex (Zhao & Xiao, 2009 ▸), including a ‘rumpled’ interface, ‘splat’-like structures and inter-splat cracks, which all may affect the stress distribution in the coating.

Currently, the most commonly used methods to measure the residual stress distribution in TBCs include the curvature method (Hsueh & Fuller, 2000 ▸; Watanabe *et al.*, 2002 ▸; Zhang *et al.*, 2013 ▸), X-ray diffraction (XRD) (Teixeira *et al.*, 1999 ▸; Mao *et al.*, 2010 ▸) and Raman spectroscopy (Mao *et al.*, 2010 ▸; Liu *et al.*, 2012 ▸). With the curvature method, only an average residual stress value through the coating can be obtained. For laboratory-based XRD (Weyant *et al.*, 2010 ▸), the penetration depth is very limited owing to the high absorption of zirconia, so measured stress values are from the sample surface. Layer-removal methods can be applied to investigate the stress distribution as a function of depth, but this is destructive (Watkins *et al.*, 1997 ▸). Raman laser light could penetrate the thin layer of zirconia, but the light will spread in the coating, making it difficult to determine the interaction volume (Liu *et al.*, 2013 ▸).

From the above description, it can be seen that, even though a good deal of work has been carried out on measuring the residual stress in TBCs, the residual stress distribution as a function of depth is seldom reported. Some research has been carried out to measure the residual stress distribution in TBCs as a function of depth by synchrotron XRD (Thornton *et al.*, 2005 ▸, 1999 ▸; Weyant *et al.*, 2010 ▸). However, either the samples were too small to represent the real stress state or only an average stress value could be obtained. *In situ* experiments were also carried out to determine the strain response of TBCs under thermal and mechanical load, and circular samples were used. The circular shape is beneficial for the application of the load (Knipe *et al.*, 2014 ▸). However, because of the cylindrical geometry, the strain measured at a greater depth always contains a component of strain at the surface.

We have used synchrotron XRD to measure the residual stress distribution in transmission geometry and reflection geometry (Li, Jacques, Chen, Daisenberger *et al.*, 2016 ▸; Li, Jacques, Chen, Xiao *et al.*, 2016 ▸). For the reflection geometry case, we developed a method to reconstruct the actual residual stress value at each depth from the average values. Until now, most of the residual stress measurements of TBCs were carried out by the well known 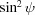 method. This method requires azimuthal diffraction data, which are usually obtained by integrating sectors of the Debye–Scherrer rings. This could result in extra data analysis after the measurement, which could be time consuming and, more importantly, induce errors during the data processing steps.

It is important to be able to measure the residual stresses from the bond coat, since they can be one of the driving forces for the ‘rumpling’ of the bond coat (Chen *et al.*, 2017 ▸). However, very few experiments have directly measured this stress distribution. Chen *et al.* (2015 ▸) measured the residual stress on the surface layer of the bond coat by a laboratory-based 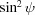 method and found a tensile stress state in the surface region of the bond coat. Zhao *et al.* (2014 ▸) also investigated the residual stress in a γ + γ′ bond coat and found the residual stress was generated from the thermal mismatch between the bond coat and the substrate. Weyant *et al.* (2010 ▸) measured the residual stresses in an NiCoCrAlY bond coat as a function of depth by synchrotron XRD. Similarly to the work of Chen *et al.* (2017 ▸), a tensile stress was found in the bond coat. Even though some work has been carried out on measuring the residual stress in NiCoCrAlY bond coats, either the samples used were too small to represent the real case or the measurement was only limited to the surface. Usually for the as-received NiCoCrAlY bond coat two phases, β and γ, are present. As far as the authors know, there are no reports on the residual stress distribution in the β phase of the as-received bond coat.

In order to resolve this problem, we used high-energy X-rays in transmission geometry from ID15 at the ESRF to penetrate through our reactively large TBC samples as a function of depth. The strains at specific depths were analysed by fitting the whole Debye–Scherer rings via *GSAS-II* (Toby & Von Dreele, 2013 ▸) to determine the degree of ellipticity and hence extract both the in-plane and the out-of-plane strains in the sample. Also, the residual strain in the β phase of the as-received bond coat was directly measured.

## 2. Experimental   

The TBCs were produced by the University West, Trollhättan, Sweden (Li, Jacques, Chen, Daisenberger *et al.*, 2016 ▸; Li, Jacques, Chen, Xiao *et al.*, 2016 ▸). The top coat was fabricated by air plasma spraying and was made of 7–8 wt% Y_2_O_3_ stabilized zirconia (∼250 µm thick). The ∼150 µm-thick NiCoCrAlY bond coat was also fabricated by air plasma spraying, and the substrate was Hastelloy X. The samples were cut to 10 × 10 × ∼5.5 mm by a slow-speed abrasive SiC cutting wheel before heat treatment in a Cabolite muffle furnace at 1423 K for 40, 91, 160 or 190 h.

**Figure 1:**
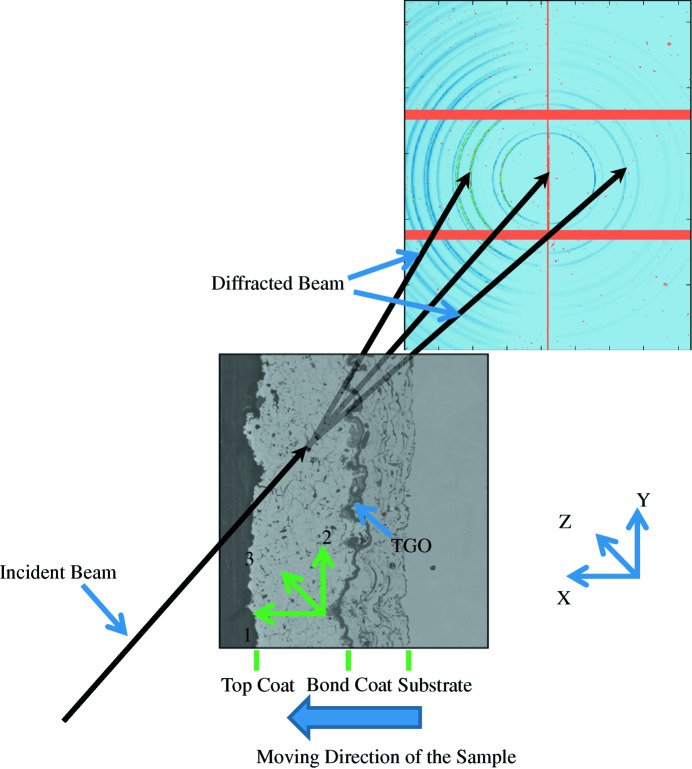
A schematic of the geometry of the residual stress measurement by synchrotron XRD. At the beginning of the measurement, the sample surface was aligned parallel to the incident beam at a grazing angle. The sample was moved perpendicular to the beam to illuminate sections through the thickness of the top coat and the bond coat. Axes 1, 2 and 3 represent the coordinates of the sample, while the *X*, *Y* and *Z* axes represent the coordinates of the equipment.

The residual strain measurement was carried out at ID15, ESRF, France. The energy of the X-ray beam used was 92.8 keV to fully penetrate the 10 mm sample, and the size of the beam was slit down to 25 × 40 µm. The experimental geometry has been described before (Li, Jacques, Chen, Xiao *et al.*, 2016 ▸) but is shown again in Fig. 1 ▸. The sample surface was aligned parallel to the beam and then moved in steps of 25 µm to ensure that the X-ray beam illuminated a straight line through the coating at a specific depth. When the beam reached the interface between the top coat and the bond coat, the Ni peak could be observed in the diffraction patterns. Since the crystal size of the substrate is quite large, the diffraction patterns became rather spotty. The exposure time for each data set was 30 s. The detector (DECTRIS Pilatus3 X CdTe 300K area detector) with 487 × 619 × 172 µm pixels in six blocks (3 × 2) was positioned about 700 and 300 mm after the sample to collect the Debye–Scherer rings from the top coat and the bond coat, respectively. The detector was positioned perpendicular to the beam with the beam pointing at the centre of the detector. A ceria powder standard was applied to calibrate the beam centre, detector tilt and sample-to-detector distance. The angle of the detector tilt was found to be 0.402°. The pseudo-strains (in units of microstrain) were found to be −181 ± 139 (in-plane strain), 108 ± 144 (out-of-plane strain) and 63 ± 119 (shear strain). Although these errors are large even at the 3σ level we are justified in saying that the pseudo-strains are quite small. After strain measurement, the samples were cross sectioned, ground and polished to 40 nm finish. The microstructure of the coating was observed by scanning electron microscopy (SEM) (FEI, QUANTA).

## 3. Strain analysis method   

The strain was analysed by the XRD^2^ method described by He & Smith (1997 ▸) using the strain fitting tool in *GSAS-II*. Generally, the stress in the coatings will distort the diffraction rings. The residual stress state in APS TBCs is considered to be a plane stress state. Thus, for the in-plane compressive stress, the diffraction rings will be extended in the in-plane direction, while for the out-of-plane direction, because of the Poisson effect, tensile strain will be present and the diffraction rings will be compressed. The relationship between strain in the sample, the sample orientation and the diffraction data is shown in the following expression:
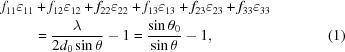
where 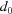 is the stress-free *d* spacing, 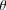 is half of the diffraction angle 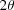, 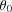 is the stress-free 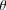 value, ∊*_ij_* are the components of the strain matrix, and 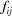 are the strains determined by the matrix operation
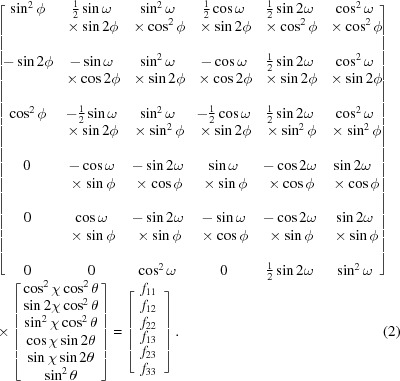
Here, 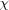 is the azimuthal angle. 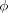 denotes the rotation angles of the specimen around the specimen surface normal (axis 1) and 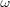 is the tilt angle of the sample around axis 3, which are both 0° in this experiment. Thus the relationship between the strain and the diffraction data can be expressed as
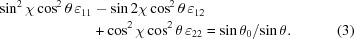
When using a high-energy X-ray beam, the value of θ can be very small; thus 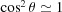 and equation (3) can be written as
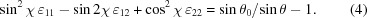
For the top coat, the 101 peak of zirconia was used to calculate the strain value, and for the bond coat, the 311 peak of Ni (γ phase) and the 211 peak of NiAl (β phase) were used to calculate the strain value.

## 4. Results and discussion   

### 4.1. Microstructure   

**Figure 2:**
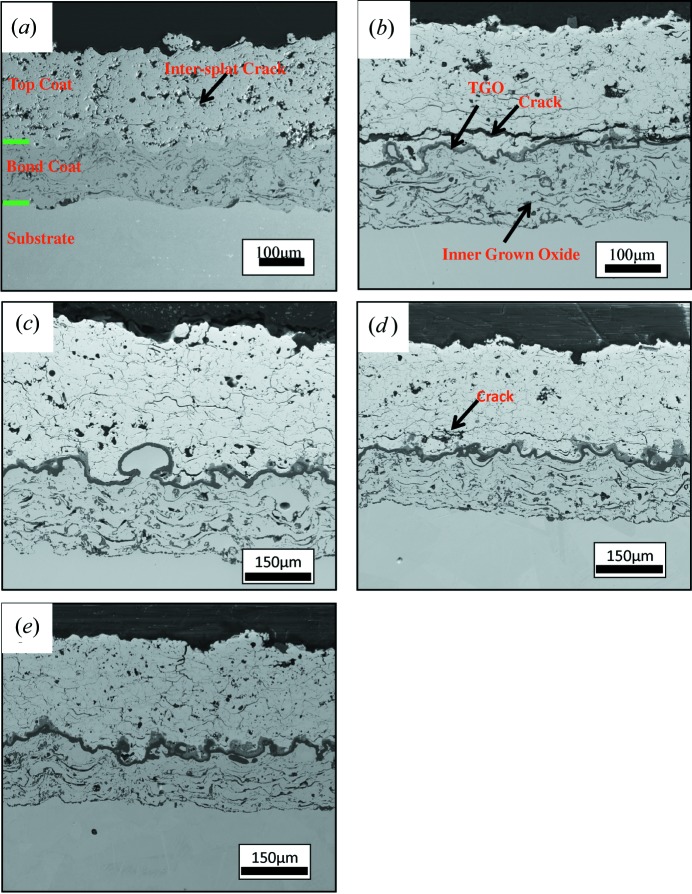
The microstructure of the as-received and the heat-treated APS TBC samples. Large cracks can be observed in samples heat treated at 1423 K for 40 and 160 h. (*a*) The as-received sample, (*b*) the sample heat treated at 1423 K for 40 h, (*c*) the sample heat treated at 1423 K for 91 h, (*d*) the sample heat treated at 1423 K for 160 h and (*e*) the sample heat treated at 1453 K for 190 h.

The microstructures of the APS TBC samples are shown in Fig. 2 ▸. From Fig. 2 ▸(*a*) it can be seen that the as-received APS TBC consists of a ‘splat’ microstructure with many inter-splat cracks and large pores, which may be due to unmelted particles during the spraying process. The splat structure can also be observed in the bond coat. No TGO was found at the interface between the top coat and the bond coat. The splat microstructure still exists in the heat-treated samples, as shown in Figs. 2 ▸(*b*)–2 ▸(*e*). The top-coat microstructures of the samples heat treated for 91 and 190 h are very similar to that of the as-received sample, despite the TGO and the inner grown oxide. However, for the samples heat treated for 40 and 160 h, a large crack can be observed in the interface region between the top coat and the bond coat. These observations imply that the existence of the large crack may not have a direct relationship with the heat-treatment time. Two layers of TGO can be observed in all the heat-treated samples. The upper layer adjacent to the top coat (with a brighter contrast) is spinel according to previous reports (Naumenko *et al.*, 2009 ▸), while the lower layer with a darker contrast is alumina, which can help to inhibit further oxidation of the bond coat. For the heat-treated sample, inner grown oxidation can be observed between the bond-coat splats. This oxidation could affect the residual strain distribution in the bond coat.

### 4.2. Residual strain distribution   

**Figure 3:**
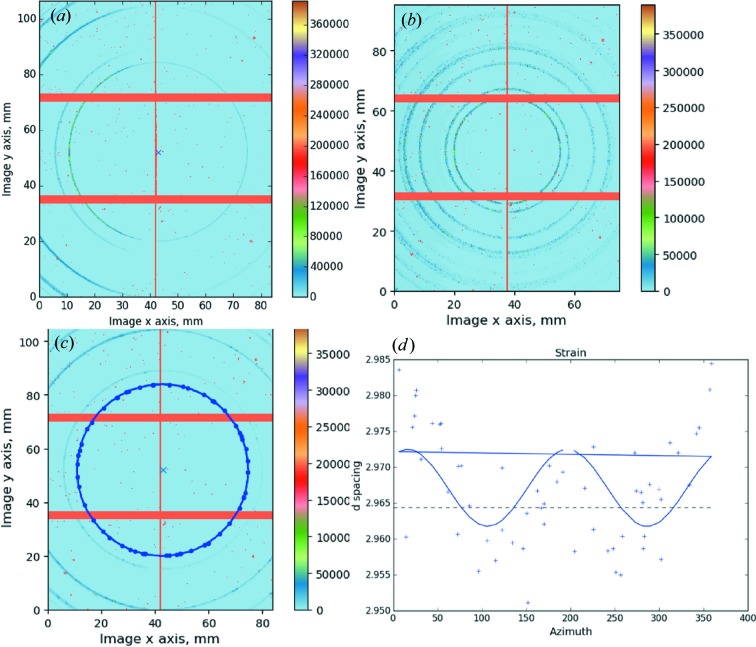
(*a*) The diffraction pattern of zirconia from the top coat. (*b*) The diffraction pattern of nickel obtained from the bond coat. (*c*) The way in which the Debye–Scherrer rings were fitted using *GSAS-II*. A reasonable fitting of the diffraction pattern can be observed. (*d*) The *d* spacing of the 101 reflection of zirconia as a function of the azimuthal angle around the Debye–Scherrer ring. The dots represent the *d* spacing obtained from the Debye–Scherrer ring and the solid line represents the fitting function for the dot data. Strain can be seen to result in a non-uniform Debye–Scherrer ring.

Typical Debye–Scherer ring patterns for the top and bond coats are shown in Figs. 3 ▸(*a*) and 3 ▸(*b*). The diffraction patterns of the zirconia were recorded at a distance of 700 mm, and thus the distance between the rings is larger. As shown in Fig. 3 ▸(*a*), one side of the rings is brighter owing to varying exit path lengths from the sample. The strain in the top and bond coats was analysed using a whole ring fit routine in *GSAS-II*. For the zirconia diffraction pattern the distance between the rings is quite large, so the pixel search range was set to 15, and for the Ni pattern [because of the small distance between the (311) and (222) rings] the pixel search range was set to 5 to inhibit misindexing. The satisfactory Debye–Scherrer ring fit of zirconia (101) is shown in Fig. 3 ▸(*c*). Fig. 3 ▸(*d*) shows the fitting of the *d* spacing of zirconia (101) at different azimuthal angles by equation (2).

**Figure 4:**
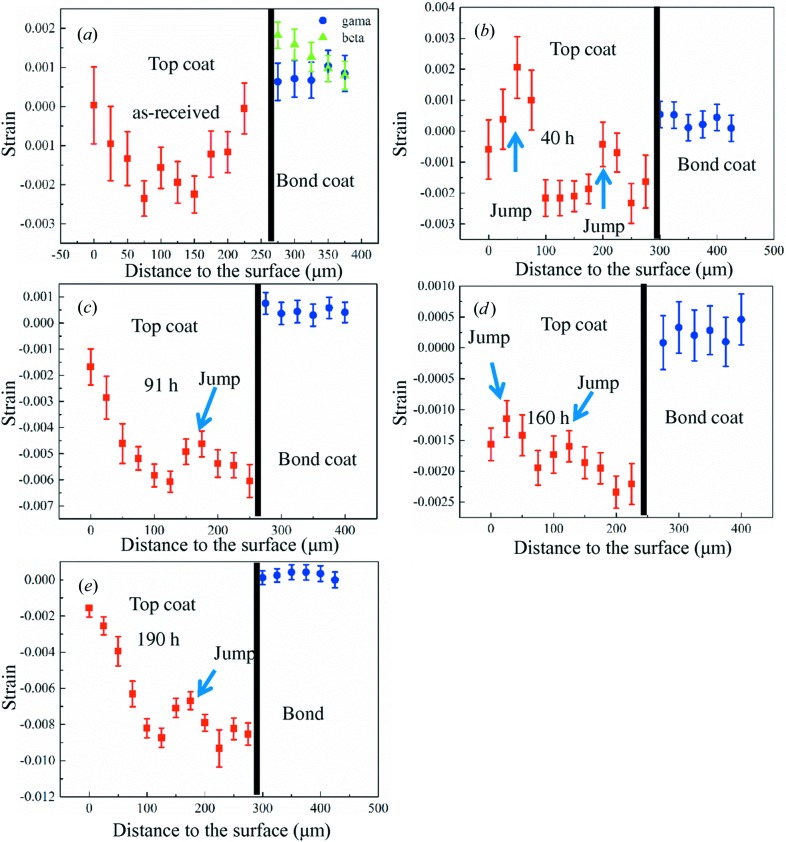
The residual strain as a function of depth in the top coat and the bond coat in the as-received sample and the samples heat treated at 1423 K. (*a*) The as-received sample, (*b*) the sample heat treated for 40 h, (*c*) the sample heat treated for 91 h, (*d*) the sample heat treated for 160 h and (*e*) the sample heat treated for 190 h. It can be seen that in all of the top coats the residual strain generally increases from the surface to the interface in a nonlinear trend, and in all of the bond coats the residual strain is generally tensile.

The measured residual strain distribution in the top and bond coats of the as-received sample is shown in Fig. 4 ▸(*a*). It can be seen that the strain levels in the top coat of the as-received sample are very low. This can be explained by the residual stresses in the as-received top coat having two contributions: firstly the quenching stresses generated during the spray process, which are tensile, and secondly the thermal stresses generated during the cooling process after thermal spraying, which are compressive. The summation of these two stresses is expected to be low and results in the small value of the residual stain. The strain distributions as a function of depth in the top coat of the samples heat treated at 1423 K for 91 and 190 h show similar trends, as shown in Figs. 4 ▸(*c*) and 4 ▸(*e*). The residual strain is generally compressive, first increasing from the surface to the interface (from around 0 to approximately −0.008), decreasing a little (around −0.006) and then increasing again to the interface (approximately −0.006). This results in a ‘jump’ feature in the trend about 100 µm from the interface. This ‘jump’ feature near the interface has been observed in all the measured samples. The samples heated at 1423 K for 40 and 160 h exhibit another ‘jump’ feature, as shown in Figs. 4 ▸(*b*) and 4 ▸(*d*). Also, the measured maximum strain values (approximately −0.002) of the samples heat treated at 1423 K for 40 and 160 h are smaller than that of the samples heat treated at 1423 K for 91 and 190 h (around −0.008 MPa). The bond coat for the as-received sample consists of two phases: β and γ. The residual strains in the two phases were therefore analysed using the 311 peak (for the γ phase) and the 211 peak (for the β phase). The results are shown in Fig. 4 ▸(*a*). It can be seen that the residual strains in the two phases are both tensile and the values are very similar (∼0.001). After heat treatment, only the γ phase is left in the bond coat. The distributions are shown in Figs. 4 ▸(*b*)–4 ▸(*e*), from which it can be seen that the residual strain is tensile and the strain values of all the heat-treated samples are approximately 0.0005. No obvious gradient for the residual strain distribution in the bond coat was observed for any of the heat-treated samples.

After a period of heat treatment at 1423 K (≥40 h), any strain distributed in the coating originating from the fabrication process should have been released. The measured strain in the top coat is therefore caused by the thermal mismatch between the top coat and the substrate. Since the coefficient of thermal expansion (CTE) of the top coat (∼11 × 10^−6^ K^−1^) is smaller than that of the substrate (∼18 × 10^−6^ K^−1^) a compressive strain state is expected in the top coat, and a tensile strain state is expected in the bond coat and the substrate. This corresponds well to our measured results for all of the samples. The surface residual stress in the TBC has been previously measured using laboratory XRD by Teixeira *et al.* (1999 ▸) and Mao *et al.* (2010 ▸). A compressive stress state has also been reported, which is similar to our result for the two large-crack-free samples shown in Figs. 2 ▸(*c*) and 2 ▸(*e*). However, the measured strain distribution as a function of depth in the top coat of these two large-crack-free samples is different from the analytical model and FEM models. From our modelling the residual strain in the top coat should be compressive and increase from the surface to the interface in a relatively uniform gradient. The main difference between our measured strain distribution as a function of depth in the top coat of the large-crack-free samples and the trend predicted by our model is the ‘jump’ feature near the interface. We note that the APS TBCs have very complex microstructures, which include the rumpled interfaces, pores and inter-splat cracks. These microstructure features could all affect the residual strain distribution in the top coat; however, we only achieve a good fit to the observed data by attributing the strain jump to the rumpled interface. The residual strain value at the interface of the sample heat treated at 1423 K for 190 h was around −0.008. This result is the same as that obtained from quantitative Rietveld refinement (Li, Jacques, Chen, Xiao *et al.*, 2016 ▸). For the samples heat treated at 1423 K for 40 and 160 h, cracks can be observed inside the top coat. Another ‘jump’ feature can be observed in the trend near the sample surface. Since the only difference between the two microstructures is the crack, we can say that this is the cause of the ‘jump’ feature. The fact that the strain measured in the samples heat treated at 1423 K for 40 and 160 h is smaller than that of the samples heat treated at 1423 K for 91 and 190 h can be explained because that part of the residual strain in the top coat has been released by the cracks.

The residual stress in the as-received sample bond coat also consists of two parts: the quenching stress and the thermal stress (Clyne & Gill, 1996 ▸). The values of both stresses are tensile owing to the thermal spray process and the CTE mismatch. For the stress distribution in the heat-treated bond coat, as discussed above, owing to the CTE mismatch, a tensile stress state is expected, which also corresponds to our measurements. Chen *et al.* (2015 ▸) measured the surface residual stress in the bond coat by laboratory XRD and found that the residual stress in the bond coat is affected by two factors: one is the CTE mismatch stress and the other is the stress induced by phase transformation from γ to β during cooling from high temperature. In our samples, as seen in Fig. 2 ▸, despite the presence of the inner grown oxide, no contrast difference within the bond coat can be observed in the backscattered electron image and only the diffraction peak of the γ phase can be observed. Thus it can be inferred that the bond coat has become a single γ phase after the diffusion process between the bond coat and the substrate and the oxidation of the bond coat. So, in our samples, the residual strain in the bond coat is caused by the CTE mismatch. As stated by Chen *et al.* (2015 ▸), after soaking the TGO off the bond coat, the residual stress at the bond-coat surface decreased by 75 MPa (∼0.000375 strain), which is the contribution of the CTE mismatch between the TGO and the bond coat. In our samples, the contribution of the residual strain comes from three parts: the CTE mismatch between the top coat and the bond coat and the substrate, the CTE mismatch between the TGO and the bond coat and the substrate, and the CTE mismatch between the inner grown oxide and the bond coat. This helps to explain why our measured value for the residual strain in the bond coat, which is purely caused CTE mismatch, is larger than that stated by Chen *et al.* (2015 ▸). Weyant *et. al.* (2010 ▸, 2002 ▸) measured the residual stress in the NiCoCrAlY bond coat and Pt aluminide bond coat using synchrotron XRD and a curvature method. They reported that the stress was tensile, which is similar to our result. The samples in this research were heat treated in a muffle furnace, which could influence the final strain distribution in the coating. But the method reported here can still be used to measure the sample subjected to a temperature gradient heat treatment. The sample used in our research is 10 × 10 × 5.5 mm, which is smaller than a turbine blade. However, the 10 mm in the *X* and *Y* dimensions should be able to ensure that the measured strain is not influenced by the edge effect and could give some indication of the residual strain distribution in a real sample.

## 5. Conclusion   

The residual strain distributions in APS TBCs after different heat-treatment times as a function of depth has been measured by synchrotron XRD and analysed using a whole Debye–Scherer ring fitting routine imbedded in *GSAS-II* (Toby & Von Dreele, 2013 ▸). The residual strain level in the as-received top coat is very low, the measured residual strain in the top coat of the heat-treated samples is compressive, and two kinds of nonlinear trends were observed. We noticed a ‘jump’ feature in the trend near the interface for all of the samples and, for some samples, another ‘jump’ feature near the surface. The difference between the two observed trends is considered to be caused by a crack in the coating. The residual strain in the β phase and the γ phase in the as-received bond coat is directly measured and it was found that both strains were tensile with a value of ∼0.001. The residual strain in the bond coat of the heat-treated samples is also tensile, with a value of about 0.001. No obvious trend of the residual strain in the bond coat is observed. These measurements have been made possible by the use of the whole-ring fitting which is significantly more efficient than the traditional reflection-by-reflection 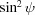 method. Note that the heat treatment in this research is carried out in a muffle furnace and the temperature in the samples is uniform, which is different from the in-service conditions of TBC with a temperature gradient. But this method could still be applied to measure the residual stress/strain distribution in TBC samples after a temperature gradient heat treatment. We deliberately chose samples of ∼1 cm^3^ (very large for tomography specimens) in order to preserve a more realistic strain distribution.
